# Discovery of a Bovine Enterovirus in Alpaca

**DOI:** 10.1371/journal.pone.0068777

**Published:** 2013-08-12

**Authors:** Shasta D. McClenahan, Gail Scherba, Luke Borst, Richard L. Fredrickson, Philip R. Krause, Christine Uhlenhaut

**Affiliations:** 1 Center for Biologics Evaluation and Research, Food and Drug Administration, Bethesda, Maryland, United States of America; 2 Department of Pathobiology, University of Illinois, Urbana, Illinois, United States of America; 3 Veterinary Diagnostic Laboratory, University of Illinois, Urbana, Illinois, United States of America; Naval Research Laboratory, United States of America

## Abstract

A cytopathic virus was isolated using Madin-Darby bovine kidney (MDBK) cells from lung tissue of alpaca that died of a severe respiratory infection. To identify the virus, the infected cell culture supernatant was enriched for virus particles and a generic, PCR-based method was used to amplify potential viral sequences. Genomic sequence data of the alpaca isolate was obtained and compared with sequences of known viruses. The new alpaca virus sequence was most similar to recently designated Enterovirus species F, previously bovine enterovirus (BEVs), viruses that are globally prevalent in cattle, although they appear not to cause significant disease. Because bovine enteroviruses have not been previously reported in U.S. alpaca, we suspect that this type of infection is fairly rare, and in this case appeared not to spread beyond the original outbreak. The capsid sequence of the detected virus had greatest homology to Enterovirus F type 1 (indicating that the virus should be considered a member of serotype 1), but the virus had greater homology in 2A protease sequence to type 3, suggesting that it may have been a recombinant. Identifying pathogens that infect a new host species for the first time can be challenging. As the disease in a new host species may be quite different from that in the original or natural host, the pathogen may not be suspected based on the clinical presentation, delaying diagnosis. Although this virus replicated in MDBK cells, existing standard culture and molecular methods could not identify it. In this case, a highly sensitive generic PCR-based pathogen-detection method was used to identify this pathogen.

## Introduction

Alpaca (*Vicugna pacos*, also known as *Lama guanicoe pacos*) are domesticated members of the New World camelid species (*Lamini*), which also include guanaco (*Lama guanicoe*), vicuna (*Vicugna vicugna*), and llama (*Lama glama*). The natural habitat for alpaca is at high altitude (3500–5000 m) in South America (Peru, Ecuador, Bolivia, and Chile) where they are kept as livestock in herds and their fiber is used much like wool. Approximately 300,000 animals [1] are in the U.S. Compared to other livestock, e.g., about 96 million cattle [2], their number is still relatively small.

Previously reported viral infections in domestic alpaca include adenovirus, equine viral arteritis virus, rabies, bluetongue virus, foot-and-mouth disease virus, bovine respiratory syncytial virus, influenza A virus, rotavirus, orf virus, bovine papillomavirus, vesicular stomatitis virus, coronavirus, bovine parainfluenza-3 virus, West Nile virus, equine herpesvirus-1 [1], [3], [4] and bovine viral diarrhea virus [5]–[13]. Bovine enteroviruses (BEV) have not previously been reported to infect alpaca. The bovine enterovirus species previously contained two types, BEV-A and BEV-B [14], [15] although a new classification structure was ratified recently, redesignating these as species *Enterovirus E* (EV-E) and *Enterovirus F* (EV-F), respectively [14], [16]. Each of the new BEV species includes multiple serotypes, with EV-E comprising four described serotypes (previously A1–4, renamed E1–E4), and EV-F containing six reported serotypes (previously B1–6, renamed F1–F6).

Recently developed approaches to virus detection have the potential to further expand understanding of viral disease in animals, including alpaca. Many of these approaches are based on non-specific PCR amplification used in conjunction with standard or high-throughput sequencing to identify PCR products.

We utilized such a method [17]–[19] to investigate an outbreak of a respiratory infection in alpaca, identifying a bovine enterovirus (EV-F), named Enterovirus F, strain IL/Alpaca, after other techniques had failed to detect any pathogen.

## Results

**Figure 1:**
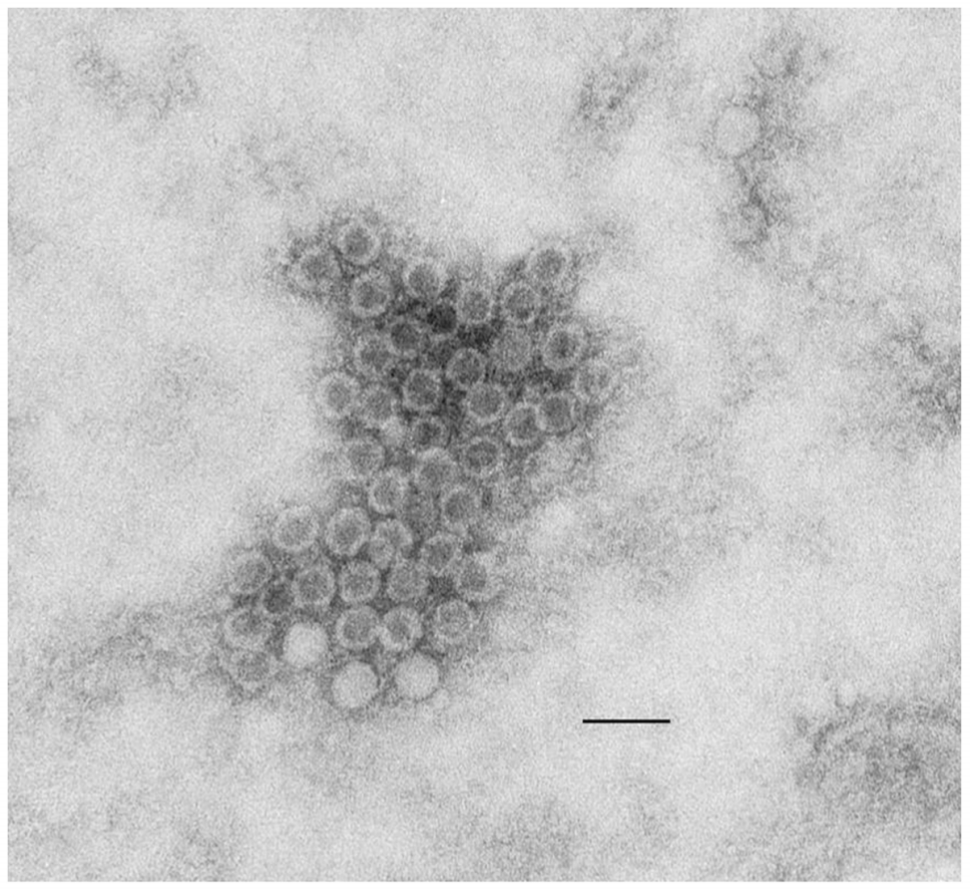
Electron photomicrograph of the alpaca virus isolate. Virus isolated from lung tissue and propagated in MDBK cell culture was imaged by negative staining EM. Virus particles are 25 to 30 nm in diameter. Image was taken at 100, 000×. Size bar is 50 nm.

Four out of 32 alpaca in an Illinois herd ranging in age from 1.5 to 14 years of age died from an acute respiratory infection (with some evidence of systemic spread in two of the animals) of unknown etiology or origin. The other animals in the herd remained clinically healthy. Necropsy revealed grossly moderate acute diffuse interstitial pneumonia in all four animals and acute renal cortical infarcts in two of the alpaca. Microscopically, marked pulmonary congestion and edema were noted in all lungs, as well as moderate erosive gastritis, acute renal infarcts, mild esophageal erosion and ulceration with suppurative esophagitis in two of the alpaca. Quantitative RT-PCR for bovine viral diarrhea virus 1 and 2 failed to detect viral genomes. A cytopathic virus was isolated on subpassage from pulmonary tissue of one affected animal using MDBK cells. Cytopathic effect (CPE) was not observed in inoculated bovine turbinate, rabbit kidney or uninoculated cells, and therefore these isolation attempts were not pursued. FITC-conjugated fluorescent antibodies against several bovine viruses (adenovirus types 1 and 5; bluetongue; bovine viral diarrhea virus; coronavirus; herpesvirus types 1, 2, and 5; parainfluenza virus 3; parvovirus; reovirus, rotavirus and respiratory syncytial virus) failed to detect a virus in the infected cell cultures. Negative staining electron microscopy (EM) of frozen and thawed infected MDBK cell culture revealed the presence of numerous, uniformly shaped, non-enveloped virus particles approximately 25 to 30 nm in diameter (Figure 1).

**Figure 2:**
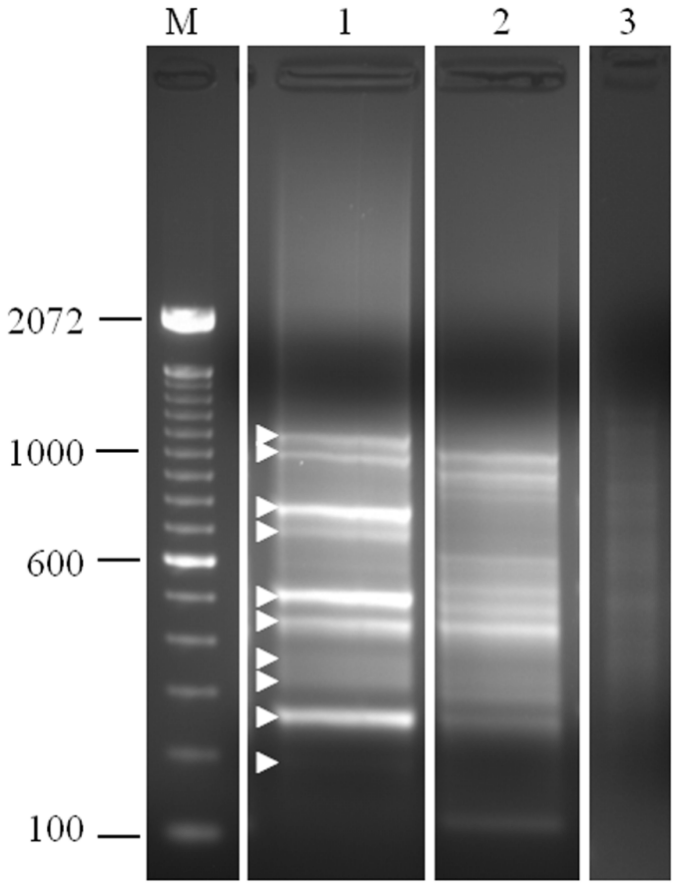
DOP-PCR products obtained from cell culture supernatant with DOP-PCR. cDNA was amplified by DOP-PCR and the obtained products were analyzed by gel electrophoresis. Marker (M): 100 bp ladder, Invitrogen, Lane 1- Infected MDBK cells, Lane 2- Uninfected MDBK cells, Lane 3- no template control. The arrowheads indicate PCR products that were sequenced and shown to be BEV-related.

In order to identify the cytopathic virus isolated from the alpaca, a generic, degenerate oligonucleotide primer (DOP) PCR-based virus detection assay [17]–[19] was utilized. Infected and uninfected cell culture supernatants were enriched for viral capsids by nuclease digestion and ultracentrifugation. Extracted nucleic acids were subjected to reverse-transcription, amplified by DOP-PCR, and separated by agarose gel electrophoresis (Figure 2). The gel electrophoresis pattern of these amplified nucleic acids differed between infected and uninfected MDBK cells. Ten bands were excised each from the infected cell lane and from the uninfected cell lane, cloned, and sequenced.

**Figure 3:**
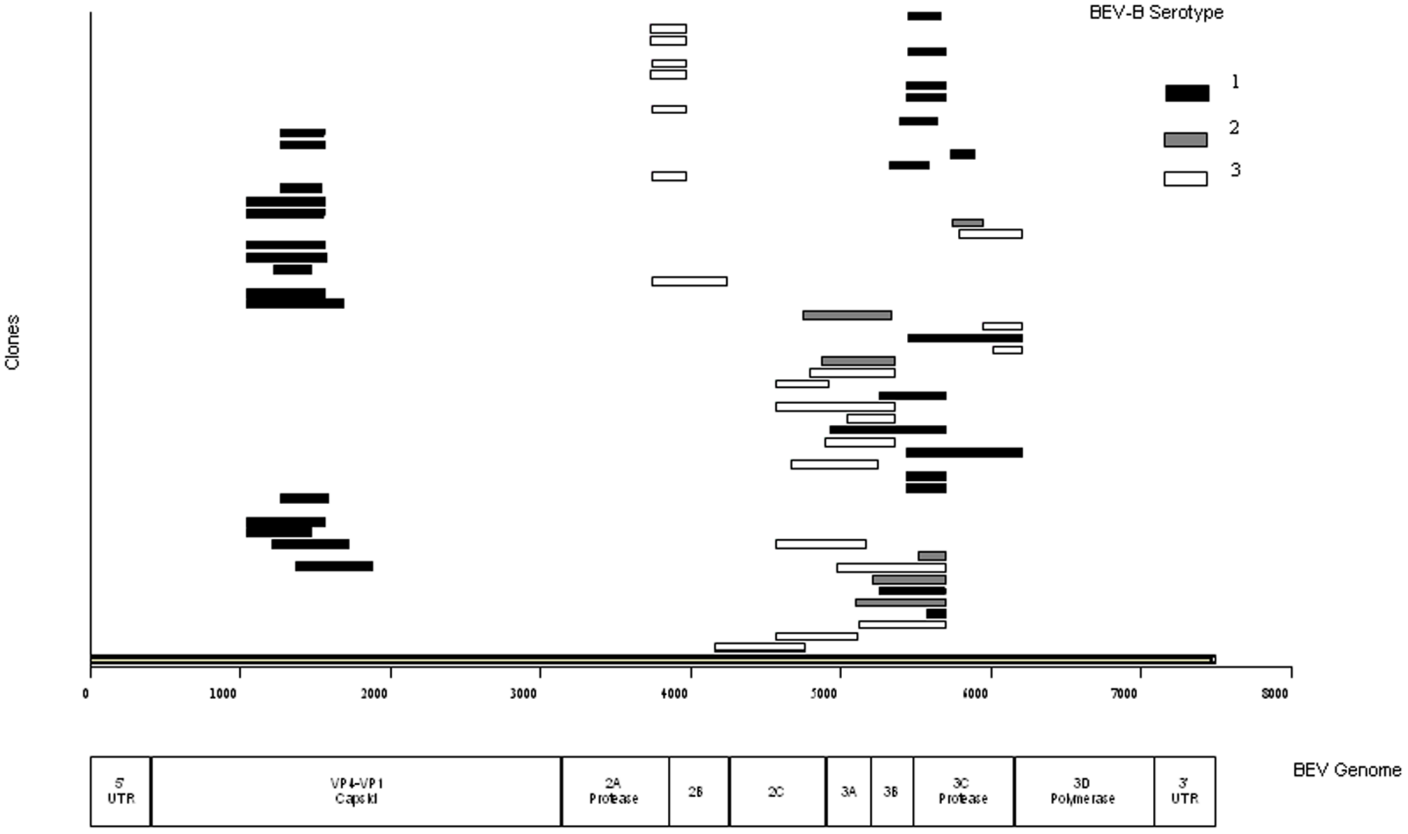
Diagram of BEV sequences identified by DOP-PCR, aligned with the EV-F genome. Sequencing of the DOP-PCR amplicons identified 47 different amplicons. These sequences had greatest homology to serotypes 1–3 of the BEV-B species, and are displayed relative to the EV-F genome. These DOP-PCR products represented 46% of the alpaca-sourced virus genome.

Sequencing of nucleic acid from the infected cell lane revealed 47 distinct products, encompassing regions with homology to approximately 46% of the EV-F genome, with sequences showing greatest homology to serotypes 1 and 3 (Figure 3). One sequence was of cellular origin due to residual MBDK cell DNA. Thirty-six distinct sequences obtained from the DOP-PCR amplicons of the negative control MDBK cells were consistent with amplification of MDBK DNA and with amplification of residual DNA in DOP-PCR reagents that we have observed previously, with no enterovirus-like sequences observed.

The remainder of the complete viral genome was identified by specific PCRs and RACE, based on primers designed from the already-obtained sequence and from BEV sequences in GenBank (Table 1). The complete 7433 bp genome for this virus, named Enterovirus F, strain IL/Alpaca, has been deposited in the GenBank database under accession KC748420.

**Table 1:** Primer sequences use for genomic sequencing and RACE PCRs of the alpaca-sourced virus.

Primer Name	Direction	Sequence	Reference
AV 1	Forward	TTT AAA ASA GYC WGG GGG TTG	
AV 1	Reverse	GTW CCG AAA GTA GTC TGT TCC	
AV 2	Forward	TGC TAA TCC CAA CCT CCG AGC	
AV2	Reverse	CGA TCA ACT GCC GTG GCA TCA G	
AV 3	Forward	GGT TAC AGT GAC AGA GTG GC	
AV 3	Reverse	GTG GGT AAA TGA GGG CAT TTC C	
AV 4	Forward	CAT CCA TGT CCA GTG TAA TGC	
AV 4	Reverse	CGT TRW AYT CVG TNK CCA TVG G	
AV 5	Forward	AGG GAA CGC CCT GAT CTA TCC	
AV 5	Reverse	CCA SWG AAC ATG MAR GTR ATC T	
AV 6	Forward	CCB ATG GMN ACB GAR TWY AAC G	
AV 6	Reverse	GTD ATD GAN GAY TGY AGC CC	
AV 7	Forward	GGG CTR CAR TCN TCH ATH AC	
AV 7	Reverse	CGA CCT TAT TCC CTG TCT GG	
AV 8	Reverse	CGA TTG TCG CAG AAT CTT TCG AC	
AV 9	Forward	CCA ACT ATG GCA TGG TTC CAT C	
AV 9	Reverse	CGT AAT CTG TAA GAC CCT GTT CC	
AV 10	Forward	GGD GAY TGY GGB GGH CTN CTY CG	
AV 10	Reverse	GTT GCC TAA GGT GCT TAA CG	
AV 11	Forward	CTT TCC CTG TTA GGA TGC TCT GG	
AV 11	Reverse	GGT GGT AGC AAG AGA CTT GC	
AV 12	Forward	GGA GCA ATT GTT CTC AAA CG	
AV 12	Reverse	GGC AAG CAC ATA CTT GGA GG	
AV 13	Forward	CCA GAT GGT AAG GAT ATG AGC C	
AV 13	Reverse	TCC CTG ACA TCC TCA GAG TCC	
AV 14	Forward	CGC TAC AAT ATC GGT AAC GTG	
AV 14	Reverse	CCA TAA AGG TGT CAT AGA CAC C	
AV 15	Forward	CGT GGT CAG ACA GGT TAC CAC	
AV 15	Reverse	CGG TGT TGC AGT TTC CAT GG	
AV 16	Forward	CAC CTT TAT GGT CTT GCC TCG	
AV 16	Reverse	CCA TCA TAA ATG CAC CCA CC	
AV 17	Forward	CCA TGG AAA CTG CAA CAC CG	
AV 17	Reverse	CCR TAY TTR TYD ATG CAC TCY TGC	
AV 18	Forward	CAT TAT GCC AAC CAG CTC AAG C	
AV 18	Reverse	CCA TCA TSA CWG GDA TYT TGC	
AV 19	Forward	CTT TGG GAA CCT CTA CAA GG	
AV 19	Reverse	GGB GGW GTC ATK ATK AGW CC	
AV 20	Forward	ATG CCH TCW GGC TGY TCD GG	
AV 20	Reverse	CCR CAR TGC CAB GCC AAT ARG C	
AV 21	Forward	GGW CTM ATM ATG ACW CCV CC	
AV 21	Reverse	TTT ACA CCC CAT CCG GYG G	
SM-9	Forward	AAYGCCCTCATTTACCCAC	
SM-10	Reverse	GACATCATCTTCAATCCACA	
SM-11	Forward	AAGAGGTATGTCGTCGTTGGCGG	
SM-17	Forward	CCACACCAGTGGGTGAAYC	
SM-18	Reverse	GACCACTGGGTGTRATATC	
SM-19	Forward	GCCAGTTTCTCACCACRG	
SM-20	Reverse	GTACCRAGCATCGCRTC	
SM-21	Forward	GATATYACACCCAGTGGTC	
SM-22	Reverse	CAGTGCTCACGGTGTGGTGG	
SM-23	Forward	GAYGCGATGCTYGGTAC	
SM-24	Reverse	GTGAAGAGTTCAAGCTTCGC	
SM-25	Forward	CCACCACACCGTGAGCACTG	
SM-26	Reverse	GCGTACAGCATGTCTTATGA	
SM-27	Forward	GCGAAGCTTGAACTCTTCAC	
SM-28	Reverse	CCARGTGCCTGTTGAGGA	
SM-29	Forward	GAACGCTCCTTGTGGTTGCC	
SM-30	Forward	CTTTAAGGGCCGATTTTGGA	
SM-31	Reverse	ATCGCCTCCCTGCGCGATG	
SM-32	Reverse	TTATTGAGGATTGCAGCCCG	
B-actin	Forward	BTCCTTCCTGGGCATGGA	[40]
B-actin	Reverse	GRGGSGCGATGATCTTGAT	[40]
GAPDH	Forward	GAAGCTCGTCATCAATGGAAA	[41]
GAPDH	Reverse	CCACTTGATGTTGGCAGGAT	[41]
Beld EV	Forward	CCCTGAATGCGGCTAA	[39]
Beld EV	Reverse	ATTGTCACCATAAGCAGCC	[39]
BEV	Forward	GGGGAGTAGTCCGACTCCG	[24]
BEV	Reverse	CAGAGCTACCACTGGGGTTGTG	[24]
N-BEV	Forward	ACGGAGTAGATGGTATTCC	[24]
N-BEV	Reverse	CGAGCCCCATCTTCCAGA	[24]
BEV-5FL	Forward	GCCGTGAATGCTGCTAATC	[24]
BEV-3-FL	Reverse	GTAGTCTGTTCCGCCTCCACC	[24]

Based on recently changed nomenclature [14]–[16], the genome of the novel virus was most closely related to EV-F (previously BEV type B) serotypes 1 and 3, with homology to EV-F complete genome sequences ranging from 75–83%. Homology with EV-E sequences was 67–68% at the genome level. Since the capsid is used for typing picornaviruses, the virus identified in this study has to be considered as type 1.

**Figure 4:**
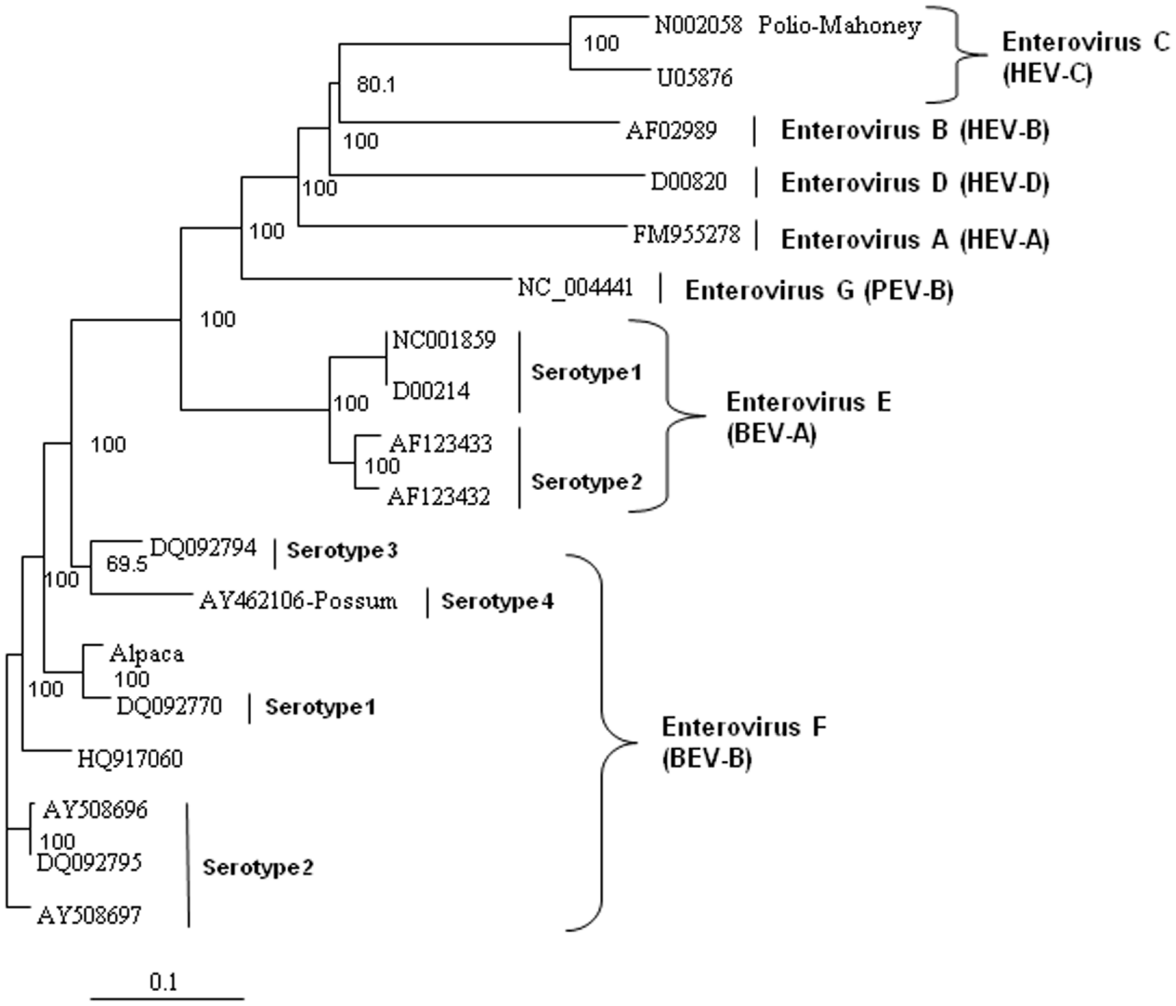
Neighbor-joining phylogenetic tree of the deduced amino acid sequences from the complete enterovirus polyprotein. Enteroviruses representing the bovine enterovirus (BEV) in species EV-E and EV-F, porcine enterovirus (PEV)/Enterovirus G, and human enterovirus (HEV)/Enterovirus A–D groups are included and species and serotypes are indicated. The amino acid sequences were aligned with the Clustal W program, and bootstrap confidence values were determined by 1000 replications. The scale bar represents the number of amino acid substitutions per site.

**Figure 5:**
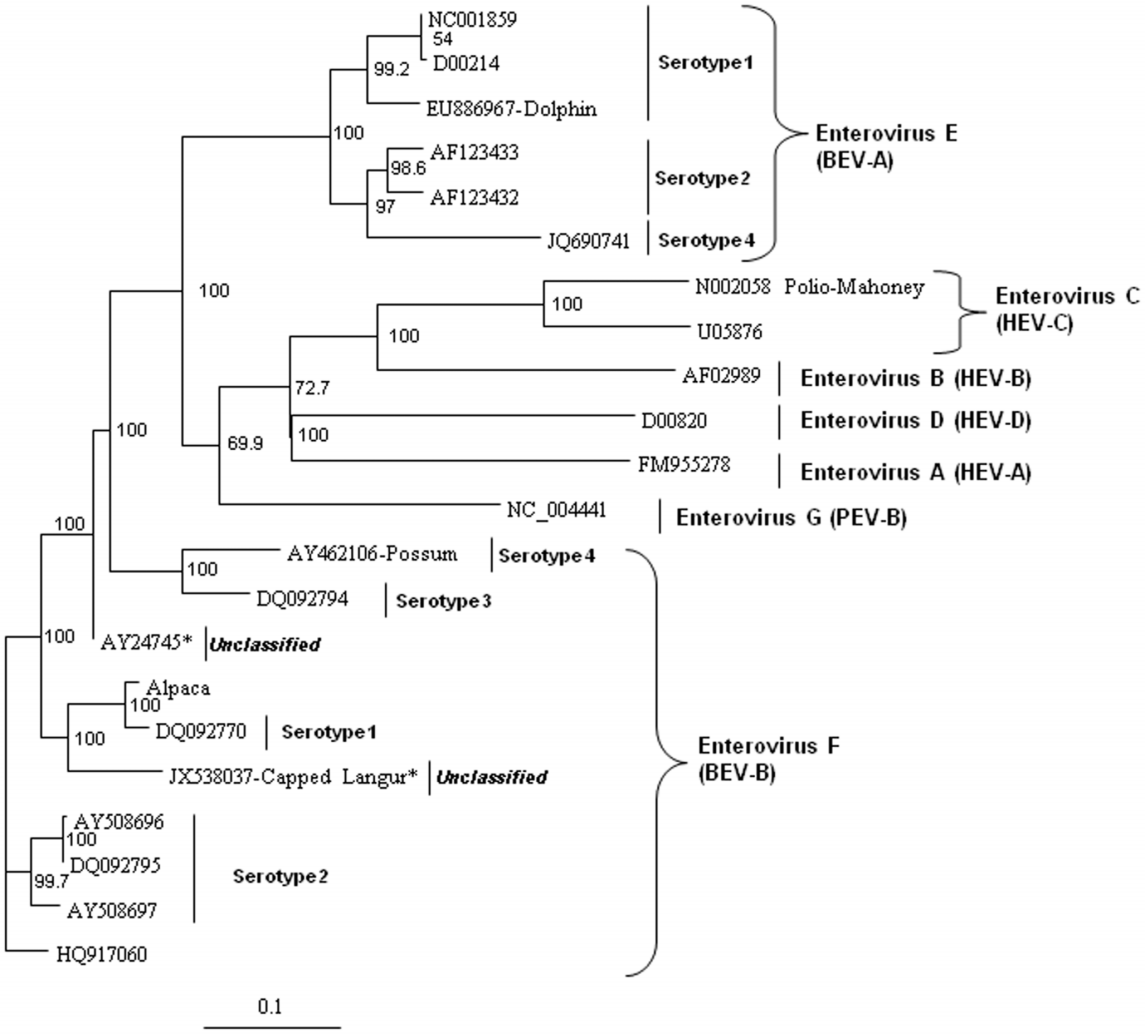
Neighbor-joining phylogenetic tree of the deduced amino acid sequences from the capsid gene. Enteroviruses representing the bovine enterovirus (BEV) in species EV-E and EV-F, porcine enterovirus (PEV)/Enterovirus G, and human enterovirus (HEV)/Enterovirus A–D groups are included and species and serotypes are indicated. The capsid sequences for the capped langur (JX538037) and unclassified AY24745 sequences (denoted with asterisks) are partial, so the correct placement of these sequences in these trees may change as more sequence data become available. The amino acid sequences were aligned with the Clustal W program, and bootstrap confidence values were determined by 1000 replications. The scale bar represents the number of amino acid substitutions per site.

**Figure 6:**
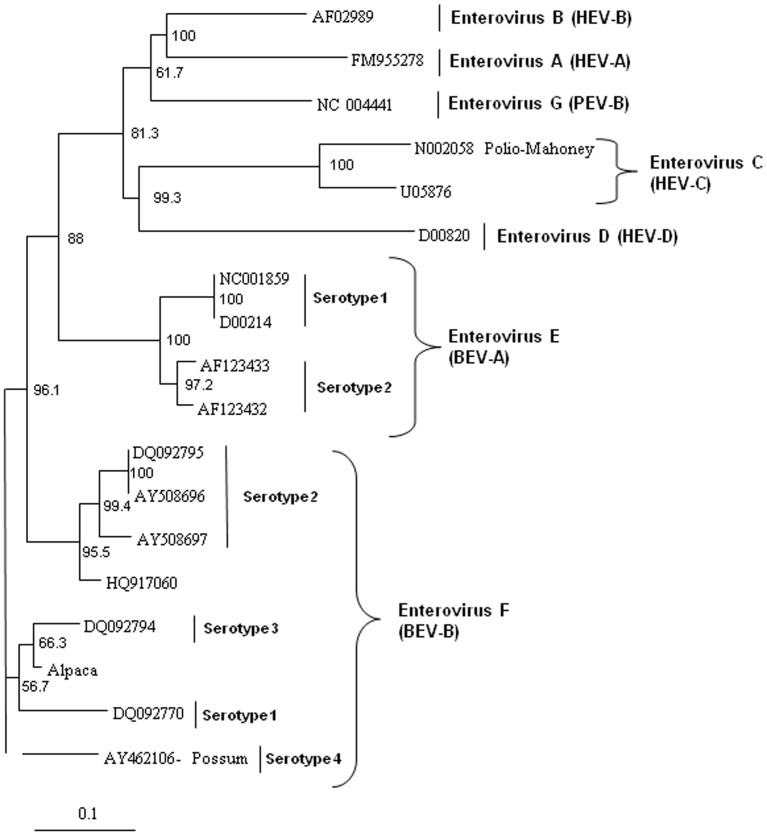
Neighbor-joining phylogenetic tree of the deduced amino acid sequences from the 2A protease gene. Enteroviruses representing the bovine enterovirus (BEV) in species EV-E and EV-F, porcine enterovirus (PEV)/Enterovirus G, and human enterovirus (HEV)/Enterovirus A–D groups are included and species and serotypes are indicated. The amino acid sequences were aligned with the Clustal W program, and bootstrap confidence values were determined by 1000 replications. The scale bar represents the number of amino acid substitutions per site.

**Figure 7:**
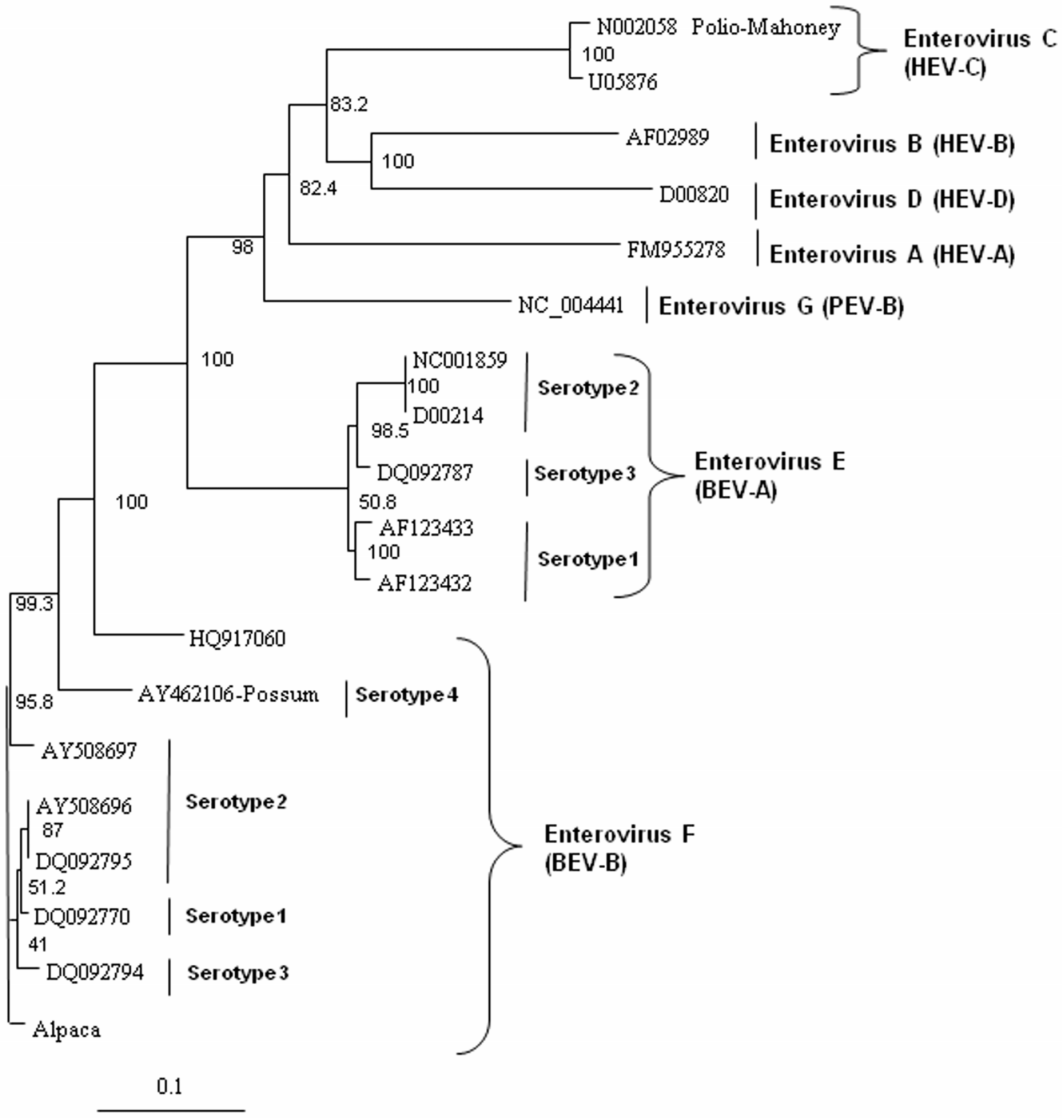
Neighbor-joining phylogenetic tree of the deduced amino acid sequences from the 3C protease gene. Enteroviruses representing the bovine enterovirus (BEV) in species EV-E and EV-F, porcine enterovirus (PEV)/Enterovirus G, and human enterovirus (HEV)/Enterovirus A–D groups are included and species and serotypes are indicated. The amino acid sequences were aligned with the Clustal W program, and bootstrap confidence values were determined by 1000 replications. The scale bar represents the number of amino acid substitutions per site.

**Figure 8:**
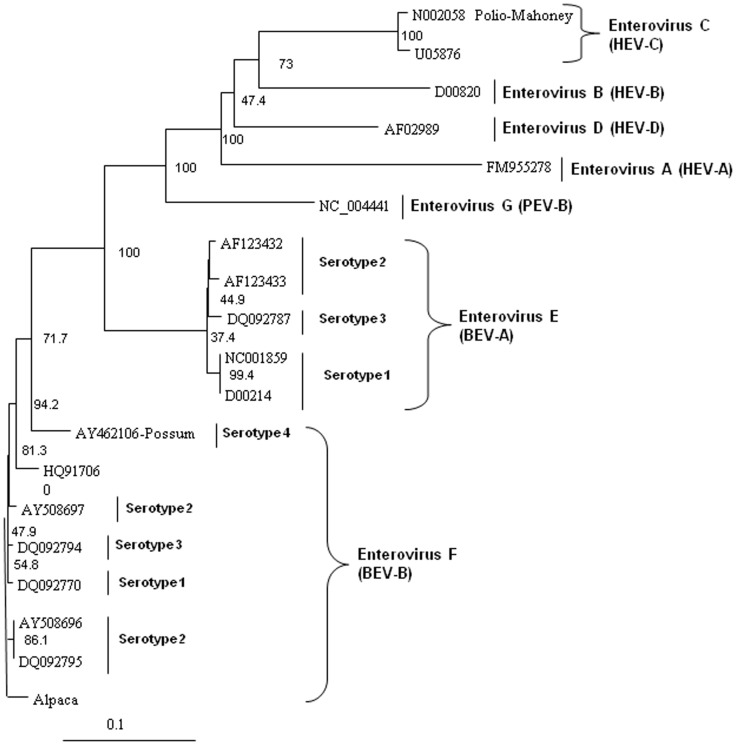
Neighbor-joining phylogenetic tree of the deduced amino acid sequences from the polymerase gene. Enteroviruses representing the bovine enterovirus (BEV) in species EV-E and EV-F, porcine enterovirus (PEV)/Enterovirus G, and human enterovirus (HEV)/Enterovirus A–D groups are included and species and serotypes are indicated. The amino acid sequences were aligned with the Clustal W program, and bootstrap confidence values were determined by 1000 replications. The scale bar represents the number of amino acid substitutions per site.

In order to analyze the virus for potential recombination and to describe it more accurately, we performed more detailed phylogenetic analyses on several proteins of the novel virus, deduced from the translated nucleic acid sequences. Analysis of the full polyprotein and the individual capsid, 2A protease, 3C protease, and polymerase proteins of the alpaca-infecting virus relative to sequences of other representative enteroviruses from bovine EV-E (BEV-A serotypes 1–4) and EV-F (BEV-B serotypes 1–4), and sequences from three unclassified EV-F viruses [16], two from bovine sources (AY724744 and AY724745) [20], and one from a capped langur (JX538037) [21], possum, porcine (PEV), and human (HEV) hosts. These analyses revealed the alpaca virus to be most closely related to EV-F (Figures 4 to 8). Based on analysis of the full polyprotein, the alpaca-sourced virus clusters most closely with the EV-F, with homologies exceeding 85%, highest with serotype 1 viruses (Figure 4), and is more distantly related to the EV-F serotypes 2 and 3, followed by the EV-E species. The more diverse capsid protein (comprising the external surface of the virus) sequence of the alpaca-sourced virus was also most closely related to EV-F, serotype 1 (Figure 5) sharing 81% and 97% identity at the nucleotide and amino acid levels respectively. The amino acid homology with serotype 2 viruses was 86–87%, and 79% with serotype 3, and 78% with a serotype 4 possum isolate. As compared with serotype 1, the capsid sequence also was less similar to the partial capsid sequences of the unclassified EV-F viruses from bovine (AY424745) and capped langur species (JX538037), each with 87% amino acid identity, although the incomplete nature of these sequences makes it impossible to be certain of the degree of relatedness. Because the capsid gene is used for serotyping picornaviruses, this virus is thus considered a type 1. However, based on 2A protease (which cleaves the viral polypeptide into its individual components) sequences, the alpaca-sourced virus groups most closely with serotype 3 with 95% homology, followed by serotype 1 with 89% homology (Figure 6), indicating that the virus had attributes of type 3 and thus could have been a recombinant between types 1 and 3. The less diverse 3C protease (which the virus also uses to cleave the polypeptide into its individual components) of the enteroviruses groups the alpaca-sourced virus most closely with EV-F, serotypes 1 and 3 (Figure 7), with 97% amino acid identity. The gene for the polymerase enzyme (which the virus uses to transcribe its RNA after infection of a cell) is also highly conserved among the enteroviruses and cannot be used to clearly delineate a serotype for the alpaca-sourced virus, which still clusters most closely with the EV-F species (Figure 8), with amino acid identity greater than 97% for serotypes 1–3 and 94% for serotype 4.

We also compared the 5′ untranslated region (UTR) of the alpaca virus with the bovine enteroviruses and found the greatest homology with EV-F strains, the highest with serotypes 1–3 and unclassified bovine sequence AY24744 at 87–90% homology. The alpaca virus homology with the EV-E 5′ UTR was 75–78%.

Several attempts to perform enterovirus-specific PCRs, using primers developed for the alpaca-source enterovirus and published EV-E and EV-F primers, were made on RNA extracted from paraffin-embedded lung tissues from the two of the alpaca (data not shown). In addition we also performed DOP-PCR on RNA extracted from these embedded tissues. Some non-specific PCR bands were evident, but sequencing of these PCR products revealed no enterovirus sequences. PCRs for the housekeeping genes β–actin and glyceraldehyde 3-phosphate dehydrogenase (GAPDH) were negative for some of the tissue samples, indicating that RNA quality was low in these fixed tissues.

## Discussion

In this report, we describe an enterovirus that was isolated on subpassage from pulmonary tissue of an alpaca that died with evidence of respiratory and systemic infection. Using a universal virus detection assay, we identified a significant portion of the genome of this picornavirus with a single PCR. This finding is consistent with the EM data that visualized non-enveloped viral particles of approximately 25–30 nm in diameter. This is the first report of a BEV isolation from alpaca.

All four of the diseased animals had similar clinical symptomatology and had similar pulmonary histology on autopsy. Because EV-F was the only potential pathogen isolated from any of these animals, the alpaca-adapted EV is a potential cause of this syndrome, although these experiments clearly did not fulfill Koch's postulates and limitations in sensitivity of the other tests that were performed do not exclude the potential for other causes. Attempts to identify EV-F by PCR of paraffin-embedded pulmonary tissue samples obtained from these animals failed. This could be due to low copy number of EV-F RNA in the sections of the paraffin blocks that were examined or low stability of the EV-F RNA under the conditions of paraffin block storage.

Enteroviruses comprise one of the nine genera of picornaviruses; all of which include members that infect vertebrates. *Picornaviridae* members are small, non-enveloped viruses with a single-stranded RNA genome of positive polarity. Members of the *Enterovirus* genus include human pathogenic poliovirus, coxsackieviruses, enteroviruses, and echoviruses. Other mammalian enteroviruses, including those infecting bovine, simian and porcine species, also have been described [22]. The only picornavirus previously reported to infect alpaca is the foot-and-mouth disease virus (FMDV), which belongs to the *Aphthovirus* genus. However, FMDV does not usually cause severe disease in alpaca [12], [23].

EV-E and EV-F are globally prevalent infections in cattle, and while virus can be shed in high titers in the feces [24], such infections are usually subclinical and their ability to cause disease in any animal is unclear. Earlier studies described enteroviruses isolated from calves suffering from respiratory disease [25]–[27]. However, in these studies, respiratory disease could not be reproduced using viral isolates from the infected calves. Subsequent studies in cattle have not been reported.

We hoped to be able to identify sequences that could account for the alpaca infection. While there are insufficient data to determine whether or not the virus adapted to alpaca, the frequent housing of alpaca with cattle without other such reports suggests that these infections are unusual. The alpaca-sourced virus has the interesting characteristic of possessing sequences that are most similar to serotype 1 (including the capsid region that is used to determine picornavirus serotype), but in at least one gene is closest to serotype 3, suggesting that this virus could have arisen by recombination of other EV-F serotypes. It is thus possible that recombination of viruses from two EV-F serotypes led to this unusual infection. The isolate has approximately 80–85% homology in its protein sequence to previously described EV-F strains, which is similar to the degree of homology shared among protein sequences from previously sequenced EV-F strains isolated from cattle, which ranges from 79–99% for EV-F strains, and 50 to 95% when EV-E strains are also considered [15]. The sequence of the alpaca-infecting virus isolate is divergent enough from previously reported strains that it does not provide clear evidence for the basis of its pathogenicity. While it is possible that this virus was transmitted directly from cattle to alpaca, it also is possible that there were one or more intermediate hosts. Besides cattle, EV-F has been reported as an infection of possum and of capped langur [28], [29]. We suspect that the absence of previously reported bovine enterovirus infections in U.S. alpaca is related to the relative isolation of alpaca herds, making it less likely that an alpaca-adapted virus would be further transmitted among alpaca.

Introducing new species (as livestock or as pets) to a habitat potentially increases the risk of an indigenous pathogen causing infections in new species. While most pathogens do not cross the species barrier due to adaptive constraints, those that succeed often cause more severe disease in the new host. Notable human examples of this phenomenon are yellow fever virus, HIV and more recently Nipah virus [30], Hendra virus [31] and SARS virus [32]. Animal examples include the devastating infections of canine distemper virus in raccoons and African lions [33]–[38]. U.S. alpaca are outside their native South American habitat and are exposed to viruses endemic to the U.S., especially those from U.S. domestic farm animals to which U.S. alpaca herds often have close proximity. Thus, recently described alpaca infections include bovine viral diarrhea virus, equine herpesvirus 1, and bluetongue virus. Newly introduced animals also can potentially carry pathogens that are relatively benign to them, but not to the indigenous fauna. The risk obviously increases if newly introduced and indigenous livestock are kept in close proximity and if their pathogens are able to remain stable in the environment or persist in the host species. Since picornaviruses are non-enveloped viruses, they often are very stable under environmental conditions, increasing the opportunity for infection of different hosts over a prolonged period of time.

While the enterovirus infection described in this report was temporally associated with illness in three other alpaca in the affected herd that may have represented limited spread of the virus, the sparse distribution of alpaca, together with the severe and rapid course of disease likely prevented further dissemination of the virus, as evidenced by the absence of other reports of similar illnesses in the herd or other alpaca in the region. However, even though it appears that this outbreak was controlled, bovine enteroviruses should be added to the list of viruses that can infect alpaca, and that could potentially be associated with severe respiratory and systemic infections in alpaca. Furthermore, considering the relative stability of enteroviruses, the ubiquity of cattle and likely frequent co-location of domestic cattle with alpaca, it is quite plausible that similar outbreaks may occur in the future. Therefore, this alpaca virus infection serves to remind us that viral species are constantly evolving and that the opportunity to infect new hosts may hasten that process.

## Materials and Methods

### Samples

Samples were obtained from animals within the affected commercial herd that had been submitted after death for a diagnostic necropsy at the University of Illinois Veterinary Diagnostic Laboratory. Grossly affected tissues were harvested at necropsy for routine histopathological examination using 10% neutral buffered formalin-fixed, paraffin-embedded, hematoxylin and eosin-stained sections. Based on the consistent gross necropsy and microscopic findings of acute diffuse interstitial pneumonia in all four alpaca, lung tissue was used for virus isolation.

### Ethics statement

The animals used in this study met the definition of “farm animals”, which are not covered by the U.S. Animal Welfare Act (9 CFR 1). Thus, IACUC or ethics committee approval was not required for these studies. The owner of the animals provided permission for these studies.

### Cell culture, virus isolation and direct fluorescent antibody detection

MDBK cells were maintained in growth media consisting of Eagle's modified essential medium (MEM; Sigma, St. Louis, MO) supplemented with a 10% horse serum (Sigma), 100 U/ml penicillin, 0.1 mg/ml streptomycin, 0.05 mg/ml gentamicin and 0.0025 mg/ml amphotericin B (Sigma) and kept in a humidified incubator at 37°C with 5% CO_2_. The following cell lines were used for virus isolation attempts: Madin-Darby bovine kidney (MDBK, ATCC CCL-22), bovine turbinate (gift from Dr. C.L. Kanitz, ADDL, Purdue University, West Lafayette, IN) and rabbit kidney (gift from Dr. C.L. Kanitz). Approximately of 10^5^ cells in one mL was plated for each cell line into separate wells of 24-well tissue culture dishes (Midwest Scientific, St. Louis, MO) containing sterile glass coverslips (12 mm, 31.5 round; Fisher Scientific, Hanover Park, IL). Approximately one gram of lung was homogenized in 10 ml of MEM containing 2× antibiotics using a Stomacher Lab Blender (Fisher Scientific). The homogenate was clarified by centrifugation at 930× g for 20 min at 4°C followed by filtration through a 0.45 µm syringe filter (Minisart NY 25; Fisher Scientific) for inoculation of 200 µl unto each of the individual cell cultures followed by the addition of 1 ml of growth media. For the inoculated cell cultures in which CPE developed, cells adherent to the coverslips were washed once in PBS, then fixed in 100% acetone for 10 min at RT followed by air drying. Appropriate FITC-conjugated anti-viral antibodies were added to the cells and incubated at 37°C for at least 30 min. Unless otherwise indicated, the following anti-viral antibodies were obtained from VMRD (Pullman, WA) and diluted 1∶4 in VMRD 4× rinse buffer: bovine adenovirus types 1 and 5; bluetongue; bovine viral diarrhea virus; bovine coronavirus; bovine herpesvirus types 1, 2, and 5; bovine parainfluenza 3 virus; bovine parvovirus; bovine reovirus; bovine respiratory syncytial virus; and bovine rotavirus (1∶30 in PBS; USDA, NVSL, Ames, IA). After incubation, the cells were washed in PBS, then overlaid with mounting media (VMRD FA mounting fluid or PBS:glycerol [1∶1]) and coverslipped. Fluorescence indicating the presence of a virus was visually determined using a Nikon Optiphot Labophot Episcopic fluorescence microscope.

### Negative staining electron microscopy

MDBK cells were grown to 90% confluency in 25 cm^2^ flasks (Midwest Scientific) containing 10 ml of growth media then inoculated with the 0.5 ml of the filtered virus isolate. After 90% CPE development, cells were harvested by freeze and thawing one time. The cell culture fluid was then clarified by centrifugation at 930× g for 20 min at 4°C. Four ml of the clarified supernatant containing 1.25% (final) neutral buffered formalin was subjected to ultracentrifugation (76 k× g for 1 hour at 4°C) to pellet the virus particles. The pellet subsequently was suspended in 100 µl of supernatant, stirred with a wooden stick and vortexed until thoroughly mixed. The sample was then placed as a drop on parafilm and a formvar plastic and carbon-coated copper grid was placed on top of the specimen droplet for 15 minutes. Excess sample was then removed with filter paper and the grid placed on 2% ammonium molybdate for 2 minutes. The grid was then dried by removing the excess fluid with filter paper, placed into a grid box and covered with drierite crystals for 10 minutes. The grid was then examined in the transmission electron microscope at 20 k and 100 k magnifications.

### Degenerate Oligonucleotide Primer- PCR (DOP-PCR)

#### Viral capsid enrichment

One mL aliquots of infected and non-infected Madin-Darby bovine kidney (MDBK) cell culture supernatant were digested with DNase I at a final concentration of 80 U/mL DNase I (2000 units lyophilized DNase I; Sigma). The lyophilized DNase I was resuspended in 200 µL DNase I dilution buffer (10 mM HEPES-KOH pH 7.9, 30 mM CaCl_2_, 30 mM MgCl_2_, 50% v/v glycerol). The digestion was carried out in 83 mM NaC_2_H_3_O_2_, 4.2 mM Mg_2_SO_4_, and 25 mM NaCl for 30 min at 37°C. The DNase digestion was stopped by adding EDTA to a final concentration of 41.5 mM. The digested samples were purified by ultracentrifugation through a 2 ml 1 M NaCl, 10 mM Tris/HCl pH 7.5 solution cushion that was overlaid with 1 ml of 10 mM Tris/HCl pH 7.5 and the digested sample. Centrifugation was carried out for 1.5 hrs, at 4°C and 64,000× g in a SW60 rotor (Beckman Coulter Inc., Fullerton, CA). The supernatant was carefully removed and the air-dried pellet was resuspended in 350 µL RLT+-buffer (Qiagen, Valencia, CA) containing 143 mM β-mercaptoethanol (MP Biomedicals Inc., Solon, OH). DNA and RNA were isolated in parallel from the same sample using the AllPrep DNA/RNA kit (Qiagen). DNA was eluted in 100 µL elution buffer; RNA was eluted in 50 µL H_2_O. RNA was transcribed in to cDNA with random hexamer primers according to the manufacturer's instructions (First Strand Kit, Invitrogen, Carlsbad, CA). Following the cDNA synthesis the RNA was digested away with 2 µL RNase H for 20 min at 37°C (First Strand Kit, Invitrogen).

#### DOP-PCR

reactions contained 1.5 mM MgCl_2_, 10 mM KCl, 10 mM Tris pH 8.4, 200 µM dNTP, 2.4 µM DOP primer 5′-CCGACTCGAGINNNNNNTGTGG-3′ (Oligos Etc., Wilsonville, OR) and 2.5 U Low DNA Taq polymerase (Applied Biosystems, Foster City, CA). The reactions were carried out with 10 µL of template. Cycling conditions consisted of initial denaturation for 5 min at 95°C; followed by 5 cycles of 1 min at 94°C, 5 min at 25°C, slow ramping at 0.1°C/sec to 30°C, 4 min at 30°C, slow ramping at 0.1°C/sec to 37°C, 3 min at 37°C, slow ramping at 0.1°C/sec to 42°C, 2 min at 42°C, slow ramping at 0.1°C/sec to 55°C, 55°C for 1 min, 72°C for 2 min; 35 cycles as follows: 94°C for 20 sec, 55°C for 1 min, 72°C for 1 min with the addition of 1 second per cycle to the extension step; final extension at 72°C for 10 min.

The DOP-PCR products were analyzed and purified by agarose gel electrophoresis. Distinct bands were excised, purified (GenElute, Sigma, St. Louis, MO), and ligated into the pCR4-TOPO® vector (Invitrogen). The ligation products were used to transform competent One Shot® TOP10 bacteria (Invitrogen) according to the manufacturer's instructions. Colony PCR was performed; these PCR products were purified (QIAquick PCR Purification, Qiagen) and sequenced using M13 primers. Sequencing was performed using a 3130×l Genetic Analyzer (Applied Biosystems, Foster City, CA). Sequences from the obtained clones were compared with the non-redundant (nr) database in GenBank using TBLASTX (NCBI, Bethesda, MD).

#### Virus genome sequencing

The sequence data obtained from DOP-PCR products as well as data for EV-E and EV-F genomes available in GenBank (NC_001859.1, AY508697.1, AY508696.1, D00214.1, AF123433.1, AF123432.1) were used to develop multiple primer pairs (Table 1) to amplify and sequence the full genome of the novel virus. PCR conditions were: initial denaturation 2 min 95°C, 40 cycles of 94°C for 30 sec, 54°C for 1 min, 72°C for 1 min, concluding with a final extension step of 72°C for 1 min. PCR products were cloned and sequenced as described above.

The 3′ end of the viral genome was sequenced using rapid amplification of cDNA ends (RACE) with a commercially available kit (Smart RACE cDNA amplification kit, Clontech, Mountain View, CA) according to the manufacturer's instructions. Viral RNA was extracted from infected cell cultures and cDNA was synthesized using MMLV reverse transcriptase and an oligo dT primer provided with the commercial kit. The cDNA was then PCR-amplified with a gene specific primer (GSP) and the universal primer provided in the kit. The GSP (SM-11) was designed based on the cloned alpaca virus cDNA sequence and the EV-E and EV-F sequences in the GenBank database and was approximately 650-bp upstream of the 3′ poly A tail. The resulting PCR products were gel purified cloned into a TA cloning vector. The 3′ viral ends were sequenced directly from the purified PCR products and from the cloned cDNA to verify the correct sequence.

### Formalin-fixed tissues

Lung tissues from alpaca and horses involved in the initial outbreak were formalin-fixed and paraffin embedded (FFPE) for future analysis and histopathology. Following the identification of enteroviruses sequences from MDBK cell cultures by DOP-PCR, we attempted to test these FFPE tissues by specific PCR assays for enterovirus sequences. Five sections of 10 µm thickness of each tissue were removed from each block with a microtome and RNA was extracted with the RNeasy FFPE Kit according to the manufacturer's instructions (Qiagen). RNA was reverse transcribed into cDNA according to the manufacturer's instructions (First strand synthesis kit, Invitrogen).

Several specific PCRs for enterovirus sequences were performed using primers designed for this alpaca sourced enterovirus (SM 9–32, Table 1) ranging from 100–500-bp and published bovine enterovirus primers [24], [39] (Beld, BEV, N-BEV; Table 1). PCRs for two bovine housekeeping genes, β–actin and glyceraldehyde 3-phosphate dehydrogenase (GAPDH) [40], [41], were also performed to verify the quality of the RNA (Table 1). Additionally DOP-PCR was performed on the cDNA as described above. PCR products were cloned and sequenced to verify the identity of the nucleic acid amplified.

### Phylogenetic analysis

The deduced amino acid sequences from the alpaca virus polyprotein containing the capsid, polymerase, and protease genes were aligned with other homologous enteroviruses available in the GenBank database. The sequences were aligned with Clustal W and neighbor-joining phylogenetic trees were constructed and viewed using Treeview [42] and Phylip software [43] with bootstrap confidence values determined by 1000 replications. GenBank accession numbers used in the analyses were: Enterovirus F, strain IL/Alpaca KC748420, BEV-A, now EV-E (serotypes 1–4) and BEV-B, now EV-F (serotypes 1–4) AF123433, D00214, AF123432, DQ092770, DQ092795, AY508696, AY508697, DQ092794, NC001859, AY462106, DQ092786, DQ092787, JQ690748, EU886967, HQ917060, NC07767, JQ690741, AY724745, and JX538037; HEV A FM955278, HEV B AF029859, HEV C U05876, and HEV D D00820; polio virus Mahoney strain N002058; and porcine enterovirus B NC_004441. Not all of the viral sequences used for analyses contained the complete polyprotein sequences, and therefore some do not appear in all of the panels of Figures 4 to 8. The capsid sequences for the capped langur (JX538037) and unclassified AY24745 sequences are partial. EV-F serotypes 5 and 6 [16] do not have nucleic acid sequences available and do not appear in these analyses.
